# Exploring the Correlation Between NLRP3 Activation and Endothelial-to-Mesenchymal Transition in the Heart of a Murine Model of Systemic Sclerosis

**DOI:** 10.3390/cells14211679

**Published:** 2025-10-27

**Authors:** Natasha Irrera, Lidia De Filippis, Davide Labellarte, Josè Freni, Giuseppe Santoro, Angelo Favaloro, Fabiana Nicita, Antonio Centofanti, Giovanna Vermiglio

**Affiliations:** 1 Department of Clinical and Experimental Medicine, University of Messina, 98125 Messina, Italy; natasha.irrera@unime.it; 2 Independent Researcher, 98125 Milan, Italy; 3 Department of Biomedical, Dental Sciences and Morphofunctional Imaging, University of Messina, 98125 Messina, Italy; davidelabellarte99@gmail.com (D.L.); jose.freni@unime.it (J.F.); giuseppe.santoro@unime.it (G.S.); angelo.favaloro@unime.it (A.F.); fabnicita@unime.it (F.N.); antonio.centofanti@unime.it (A.C.); 4 Department of Oral and Maxillofacial Sciences, Sapienza University of Rome, 00185 Rome, Italy

**Keywords:** SSc, EndMT, heart, vessels, oxidative stress, inflammation

## Abstract

Systemic sclerosis (SSc) is an autoimmune disease marked by fibrosis in various organs, including the heart. Cardiac involvement influences prognosis, but the underpinning mechanisms remain unclear. This study investigates myocardial changes in a murine SSc model induced by subcutaneous injection of HOCl, with a specific focus on alterations in structural proteins and inflammatory markers, oxidative stress, and vascular remodeling. Hearts were collected from SSc mice after 6 weeks, and structural, inflammatory, and oxidative stress markers were evaluated by Hematoxylin and Eosin (H&E) staining, Masson’s Trichrome, and immunohistochemical analysis. Increased vimentin and α-SMA expression were detected in the vasculature, indicating endothelial dysfunction and myofibroblast activation, alongside a decrease in CD31 expression, consistent with an endothelial-to-mesenchymal transition (EndMT). Concomitant increases in macrophage (CD68, F4/80, EP29, EPR1) and inflammasome markers (Caspase-1, IL-1β and NLPR-3) were observed together with a remarkably augmented level of MMP3, MMP9, Collagen I and TGF-β, thus suggesting that inflammation and matrix remodeling correlate with endothelial dysfunction. Accordingly, the increased levels of NRF2 and HOMX1 suggested a compensatory protective response against oxidative stress. These data suggest that both immune cell- and inflammasome-mediated inflammation signaling play a key role in endothelial dysfunction by altering the balance between fibrosis and vascular remodeling markers.

## 1. Introduction

Systemic sclerosis (SSc), also known as scleroderma, is a rare autoimmune disease characterized by immune system activation, widespread microvascular injury, and progressive fibrosis of the skin and multiple internal organs, including lungs, kidneys, and heart [1,2,3]. Among these, cardiac involvement is particularly insidious, often remaining clinically silent until advanced stages, where it significantly worsens the prognosis. Cardiac manifestations range from conduction abnormalities and arrhythmias to myocardial fibrosis, diastolic dysfunction, and right heart failure, frequently culminating in pulmonary arterial hypertension (PAH) [4,5,6].

Despite growing recognition of the clinical impact of cardiac complications in SSc, the underlying molecular mechanisms of myocardial involvement remain incompletely understood. A major limitation is the scarcity of direct histopathological data, due to the invasive nature of endomyocardial biopsy and the challenge of distinguishing primary SSc-related fibrosis from changes secondary to common cardiovascular comorbidities [7]. As a result, most current knowledge derives from indirect methods, such as imaging or post-mortem analysis, which do not capture early, cell-specific pathogenic events [8]. Understanding how immune dysregulation, vascular injury, and inflammatory signaling converge to initiate myocardial fibrosis remains a critical gap.

Inflammation plays a pivotal role in the pathogenesis of SSc, both systemically and within target organs [9,10,11]. Recent studies have highlighted the involvement of the NLRP3 inflammasome, a key component of the innate immune system that senses cellular stress and danger signals. Upon activation, NLRP3 promotes the maturation of pro-inflammatory cytokines, including interleukin-1β (IL-1β), thereby contributing to tissue damage and fibrosis [12,13,14]. In models of lung and kidney fibrosis, NLRP3 has been shown to regulate epithelial-to-mesenchymal transition (EMT) [14] as well as endothelial-to-mesenchymal transition (EndMT), a process whereby endothelial cells lose their native phenotype and shift to a mesenchymal, fibrogenic profile [15]. While EndMT is increasingly recognized as a contributor to fibrotic remodeling in SSc-affected tissues, its involvement in the heart, and particularly its regulation by inflammasome activation, remains poorly explored. Moreover, oxidative stress, driven by excessive reactive oxygen species (ROS) and impaired antioxidant defenses, is a key amplifier of endothelial dysfunction and fibrosis in SSc [16,17]. ROS also act as potent activators of the NLRP3 inflammasome, creating a feed-forward loop that sustains chronic inflammation, fibroblast activation, and collagen deposition [18]. Nevertheless, the combined contribution of oxidative stress and inflammasome signaling in promoting EndMT and myocardial fibrosis in SSc has not yet been thoroughly investigated.

To address this gap, we explored the relationship between NLRP3 inflammasome activation, oxidative stress, and EndoMT in the heart by using hypochlorous acid (HOCl)-induced murine models of SSc, providing groundbreaking insights into the pathophysiology of cardiac fibrosis and identifying potential therapeutic targets.

## 2. Materials and Methods

### 2.1. Animals

Samples obtained from eight female BALB/c mice (Charles River, Calco, Italy), aged 6 weeks, were used in this study. The animals were housed in the Animal Facility of the University of Messina under a controlled environment, with free access to a standard diet and water ad libitum. The project received approval from the Ethics Committee of Messina University as well as from the National Ethics Committee for Research Animal Welfare of the Italian Ministry of Health (authorization no. 428/2022-PR). All experimental procedures were conducted in accordance with: (1) the ARRIVE guidelines [19]; (2) the Italian Guidelines for the Care and Use of Laboratory Animals (D.L.116/92); and (3) the European Directive (2010/63/EU).

Seven animals were daily injected with HOCl [20] for six weeks to induce SSc, while the remaining seven animals received a saline solution injection for the same period (Vehicle group).

### 2.2. Heart Tissue Collection from Mice

Hearts were collected from the HOCl and vehicle groups after six weeks. The hearts were carefully dissected free of surrounding tissues and immediately fixed in 10% buffered formalin for 24–48 h at room temperature. After fixation, the hearts were washed with phosphate-buffered saline (PBS) and then transferred to 70% ethanol for long-term storage at 4 °C until further processing.

### 2.3. Sample Processing

After fixation, the hearts were dehydrated through a graded series of ethanol solutions (70%, 95%, and 100%) for 1 h each, followed by two changes in xylene for 1 h each, to remove alcohol. The tissue was then infiltrated with paraffin wax using a tissue processor (Leica TP1020, Leica Biosystems Nussloch GmbH, Nussloch, Germany) according to standard protocols. Once infiltration was complete, the hearts were embedded in paraffin blocks, orienting the tissue to enable optimal sectioning of the myocardium. The blocks were left to solidify at room temperature. Paraffin-embedded heart tissue was sectioned into 5 µm thick slices by a microtome (Leica RM2135). Sections were mounted on glass slides, ensuring that the myocardial tissue was correctly oriented. The slides were placed in an oven at 60 °C for 1 h to ensure adequate tissue adherence to the glass surface.

### 2.4. Histological Staining

For histopathological evaluation, the sections were deparaffinized by immersion in xylene and subsequently rehydrated by using a descending series of ethanol solutions (100%, 95%, 70%, and 50%), followed by washing in distilled water. The stains performed are presented below.

#### 2.4.1. Hematoxylin and Eosin (H&E) Staining

Tissue sections were stained with Hematoxylin for five minutes and subsequently rinsed in running tap water. Eosin was then applied for a period of two minutes, after which the slides were rinsed once more in water and dehydrated through ascending concentrations of ethanol (70%, 95% 100%). Following dehydration, the slides were cleared in xylene and mounted with a coverslip through an Eukitt mounting medium.

#### 2.4.2. Masson’s Trichrome Staining

Following the rehydration process, tissue sections were stained using Masson’s Trichrome method to evaluate collagen deposition. This procedure was executed in accordance with established protocols, wherein the tissue sections were initially stained with Weigert’s hematoxylin, subsequently followed by Biebrich scarlet acid fuchsin, phosphotungstic acid, and aniline blue to accentuate the collagen fibers. The prepared slides were then dehydrated, cleared, and mounted appropriately.

### 2.5. Immunohistochemistry (IHC)

Immunohistochemical staining was conducted to evaluate the expression of α-SMA, CD31, Vimentin, Nos, and Nrf2. The sections underwent deparaffinization and rehydration and were then subjected to antigen retrieval by boiling in a citrate buffer (pH 6.0) for 10 min. Following cooling, the sections were treated with 5% bovine serum albumin (BSA) in phosphate-buffered saline (PBS) for one hour to prevent non-specific binding. Primary antibodies (Table 1), were meticulously applied to the sections and allowed to incubate overnight at a temperature of 4 °C. After the incubation with primary antibodies, the sections were treated with the appropriate secondary antibody conjugated to horseradish peroxidase (HRP) for one hour at room temperature. Immunoreactivity was visualized using 3,3-diaminobenzidine (DAB) as a chromogen, and the sections were counterstained with hematoxylin. The following kit was used: R.T.U. Vectastain Universal (Vector Laboratories, Inc., Burlingame, CA, USA).

### 2.6. Immunofluorescence

For immunofluorescence, sections were subjected to deparaffinization and rehydration as previously described. Following an hour of blocking with 1% Bovine Serum Albumin (BSA) and 0.3% Triton-X100 in Phosphate-Buffered Saline (PBS), the tissue sections were incubated with primary antibodies (Table 1) overnight at 4 °C. After the primary antibody incubation, sections were washed in PBS and incubated with fluorophore-conjugated secondary antibodies (Table 2) for one hour at room temperature while protected from light. Thereafter, the sections were again washed in PBS, mounted with ProLong Gold antifade reagent that contained 4′,6-diamidino-2-phenylindole (DAPI) for nuclear staining, and covered with coverslips.

### 2.7. Microscopy and Image Analysis

The Hematoxylin–Eosin and Masson’s staining slides were observed with a Nikon Ci-L (Nikon Instruments, Tokyo, Japan) light microscope, and the micrographs were obtained using a digital camera (Nikon Ds-Ri2) and saved as Tagged Image Format Files (TIFF) with the Adobe Photoshop CS5 12.1 software. The immunofluorescence reactions were observed with a Zeiss LSM 510 DUO (Carl Zeiss, Jena, Germany) confocal laser scanning microscope. All photos were digitized at a resolution of 8 bits into an array of 2048 × 2048 pixels. Optical sections of fluorescent specimens were acquired using a Helium–Neon (HeNe) laser (Carl Zeiss, Jena, Germany) with a wavelength of 543 nm at a scanning speed of 62 s and up to 8 averages. Sections were achieved using a pinhole of 250 μm. Contrast and brightness were determined by analyzing the most luminously labeled pixels and selecting the parameters that facilitated clear visualization of structural features while preserving the maximum pixel intensity (~200). Digital images were cropped, and figure montages were assembled using Adobe Photoshop 12.1. The fluorescence and immunoenzymatic pattern intensity of all tested proteins and the Masson staining quantification were measured using ImageJ software 1.54p (National Institutes of Health, Bethesda, MD, USA) [21], and the resulting numerical data were subjected to statistical analysis. To ensure accuracy, the intensity values were measured across five microscopic fields for each antibody. Analyses were normalized to the total tissue area and carried out by investigators blinded to the experimental groups to minimize bias.

### 2.8. Statistical Analysis

All data are presented as mean ± standard deviation (SD). Data normality was assessed using the Shapiro–Wilk test. As the data met the assumption of normality, comparisons between vehicle and HOCl groups were carried out using an unpaired *t*-test with Welch’s correction. The analyzed parameters included the fluorescence intensity of the following markers: α-sarcoglycan, γ-sarcoglycan, dystrophin, β-dystroglycan, talin, vinculin, CD11B, EP29, EPR1, F4/80, Ly6G, MMP-3, MMP-9, Caspase-1, IL-1β, and NLRP-3. The same test was applied to compare the immunoenzymatic staining intensity of α-SMA, CD31, NRF2, NOS, vimentin, Collagen I, TGF-β, and HOMX1 between the two groups. Statistical analyses were performed using SPSS for Windows, version 26.0 (IBM, New York, NY, USA). A *p* < 0.05 was considered statistically significant. Graphs were generated using GraphPad Prism (version 10), which displays the corresponding statistical results.

## 3. Results

### 3.1. Heart Structure Evaluation

**Figure 1:**
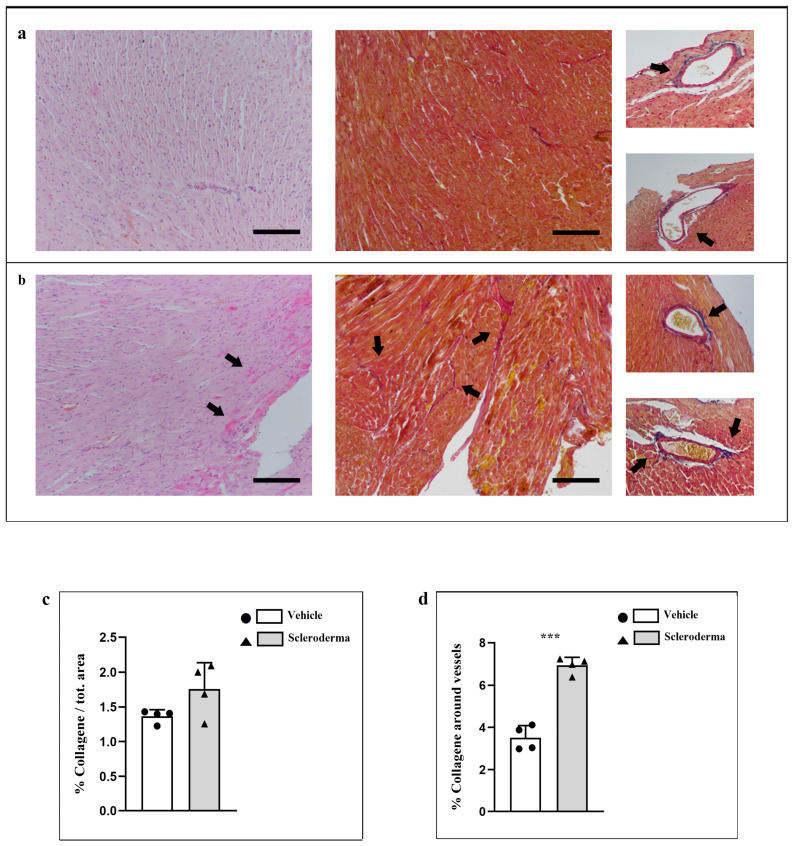
Compound panel of H&E- and Masson’s Trichrome-stained sections of hearts from vehicle (panel (**a**)) and HOCl (panel (**b**)) mice. Magnification 20×, black scale bar 50 µm. Semi-quantification of blue staining across the section and around the vessels (panels (**c**,**d**)). The graphic shows a significant increase in collagen fibers around vessels in HOCl mice (*** *p* < 0.001) (panel (**d**)).

The data acquired through H&E staining revealed a healthy structure of the ventricular wall in the vehicle group, characterized by homogeneous organization of cardiomyocytes and subtle connective septa interspersed among cardiomyocytes (Figure 1, panel a). In the context of the HOCl heart, certain regions exhibited particularly pronounced staining, likely indicative of fibrillar accumulation amid the cardiomyocytes, although no conclusive evidence of fibrosis was detected (Figure 1, panel b). These areas showed more prominent septal structures when compared to the vehicle group (black arrows), notwithstanding the absence of definitive signs of fibrosis. Masson’s trichrome staining has further corroborated the observations obtained by H&E and allowed us to enhance visualization of connective tissue between cardiomyocytes (Figure 1, panels a,b, black arrows). Masson’s Trichrome staining accentuated a distinct bluish hue in the connective tissue enveloping the vessels in the scleroderma heart, as a sign of early perivascular collagen deposition (Figure 1, panel b, black arrows). This perivascular staining was considerably pronounced in comparison to the mild staining observed in the vehicle control hearts (Figure 1, panel a, black arrows), thus suggesting the early stages of an extracellular matrix remodeling fibrotic response within the scleroderma-myocardium. All these data were supported by semi-quantification of blue staining across the section and around the vessels (Figure 1, panels c,d).

### 3.2. Immunofluorescence Analysis of Structural Proteins and Inflammatory/Pro-Fibrotic Markers

**Figure 2:**
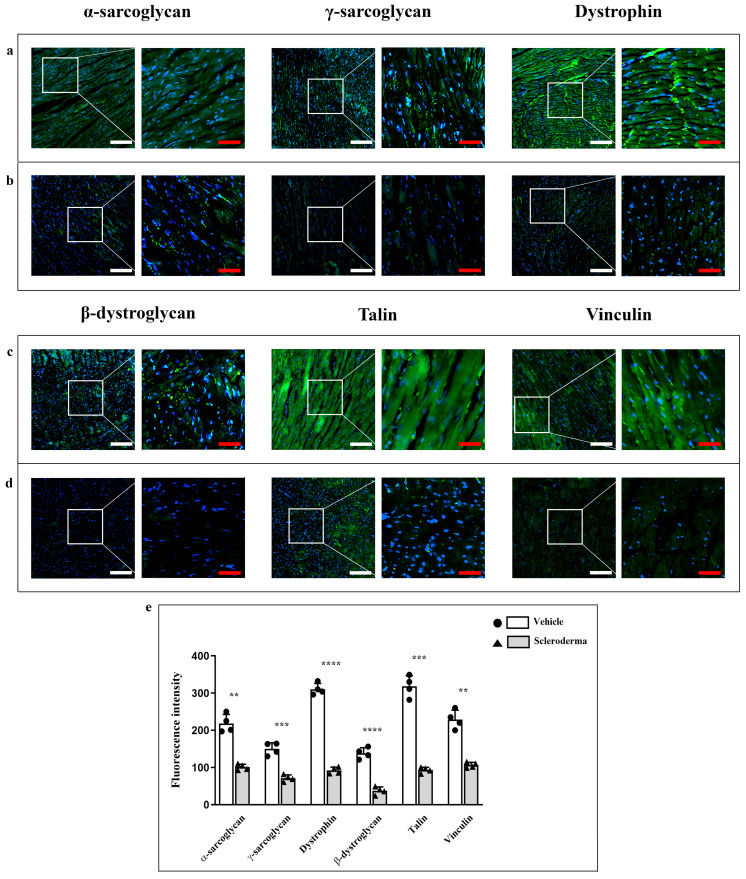
Compound panel of immunofluorescence images for the evaluation of α-sarcoglycan, γ-sarcoglycan, dystrophin, β-dystroglycan, talin, and vinculin expression in heart sections obtained from vehicle (panels (**a**,**c**)) and HOCl (panels (**b**,**d**)) mice. The graphic shows a significant reduction in all markers in HOCl mice (** *p* < 0.01, *** *p* < 0.001, **** *p* < 0.0001) (panel (**e**)). Magnification 20×, white scale bar 50 µm; magnification 40×, red scale bar 25 µm.

Immunofluorescence reactions revealed positivity in cardiomyocytes for the following antibodies in the vehicle-treated heart: α-sarcoglycan, γ-sarcoglycan, dystrophin, β-dystroglycan, talin, and vinculin (Figure 2, panels a,c). In the HOCl heart, a significant reduction in fluorescence signal was noted for all proteins (Figure 2, panels b,d) in comparison to the controls (vehicle group). The fluorescence intensity values were reported in a graphic confirming the reduction in α-sarcoglycan, γ-sarcoglycan, dystrophin, β-dystroglycan, talin, and vinculin expression (Figure 2, panel e).

**Figure 3:**
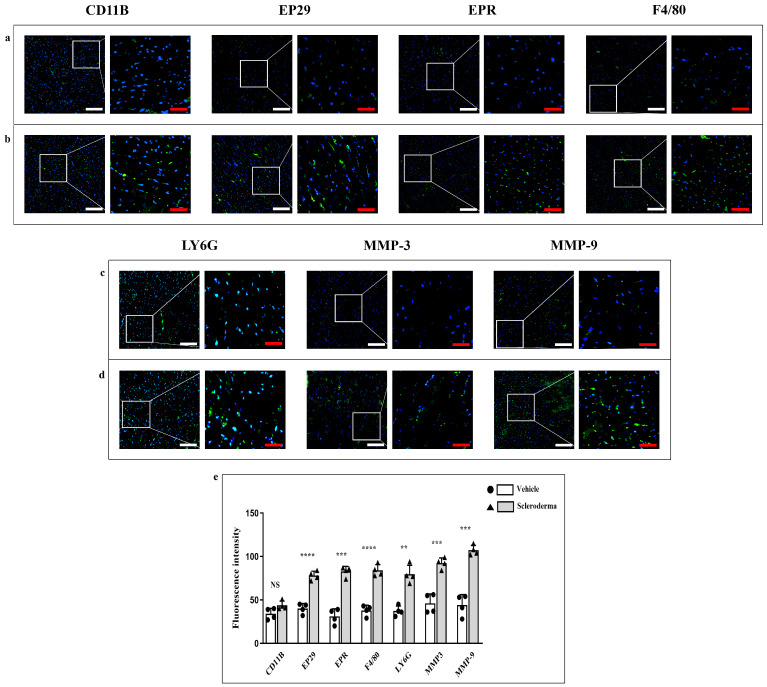
Compound panel of immunofluorescence images for the evaluation of CD11B, EP29, EPR1, F4/80, Ly6G, MMP-3, and MMP-9 expression in heart sections from vehicle (panel (**a**,**c**)) and HOCl (panel (**b**,**d**)) mice. The graphic shows a significant increase in all markers in HOCl mice (** *p* < 0.01, *** *p* < 0.001, **** *p* < 0.0001) except for CD11B (not significative, NS) (panel (**e**)). Magnification 20×, white scale bar 50 µm; magnification 40×, red scale bar 25 µm.

Immunofluorescence reactions by using antibodies against CD11b, EP29, EPR1, F4/80, Ly6G, MMP3, and MMP9 exhibited a basal fluorescence pattern in the vehicle heart (Figure 3, panels a,c). The fluorescence pattern for each marker significantly increased in the HOCl heart (Figure 3, panels b,d), thus indicating an upregulation of the inflammatory markers and matrix remodeling enzymes in response to the disease. The fluorescence intensity means values for each marker are shown in the graphic (Figure 3 panel e). The heightened expression of these markers denotes an intensified inflammatory response and tissue remodeling within the myocardium of HOCl mice.

**Figure 4:**
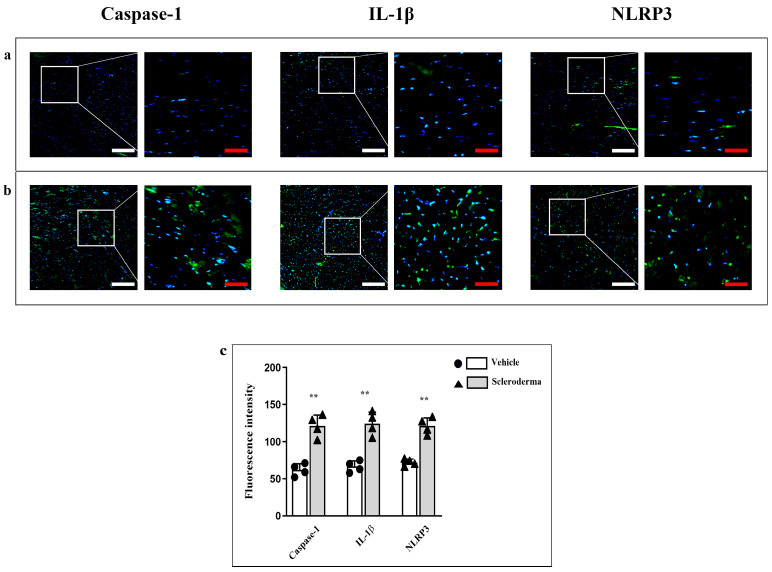
Compound panel of immunofluorescence images for the evaluation of Caspase-1, IL-1β, and NLRP-3 expression in heart sections obtained from vehicle (panel (**a**)) and HOCl (panel (**b**)) mice. The graphic shows a significant increase in Caspase-1, IL-1β, and NLRP-3 expression in SSc mice (HOCl group; ** *p* < 0.01) (panel (**c**)). Magnification 20×, white scale bar 50 µm; magnification 40×, red scale bar 25 µm.

Moreover, immunofluorescence reactions of the NLRP3 inflammasome pathway and, in detail, of NLRP-3, Caspase-1 and IL-1β showed a high fluorescence staining pattern of all these markers in HOCl group if compared to the vehicle group where the fluorescence was faintly detectable (Figure 4a,b). The fluorescence intensity means values are shown in the graphic (Figure 4c).

### 3.3. Immunoenzymatic Analysis

**Figure 5:**
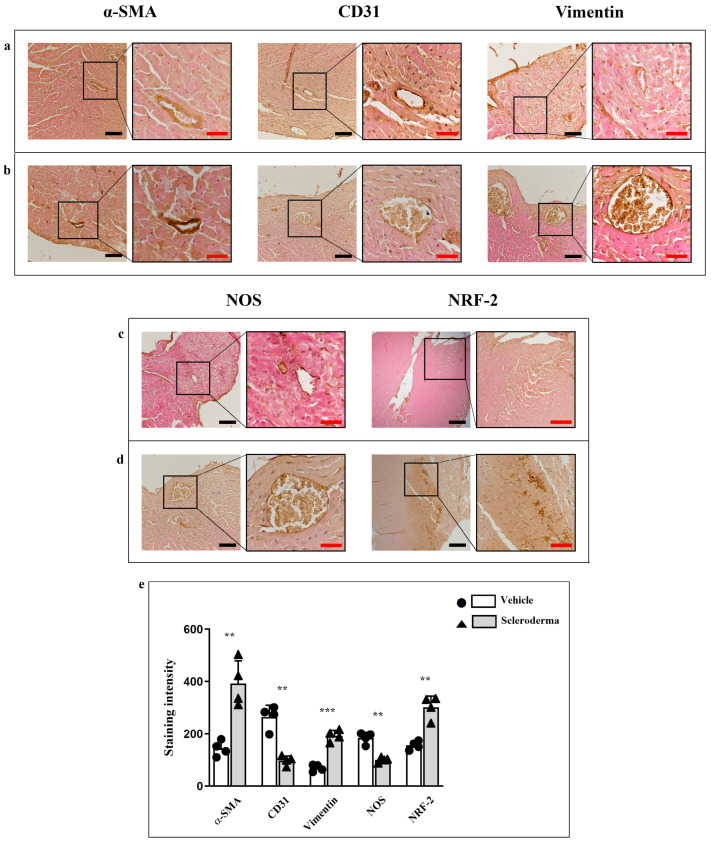
Compound panels of immunoenzymatic images for the evaluation of alpha-SMA, CD31, NRF2, NOS and vimentin in heart sections obtained from vehicle (panels (**a**,**c**)) and HOCl mice (panels (**b**,**d**)). The graphic shows the staining intensity and the statistical difference between groups (** *p* < 0.01, *** *p* < 0.001) (panel (**e**)). Magnification 20×, black scale bar 50 µm; magnification 40×, red scale bar 25 µm.

**Figure 6:**
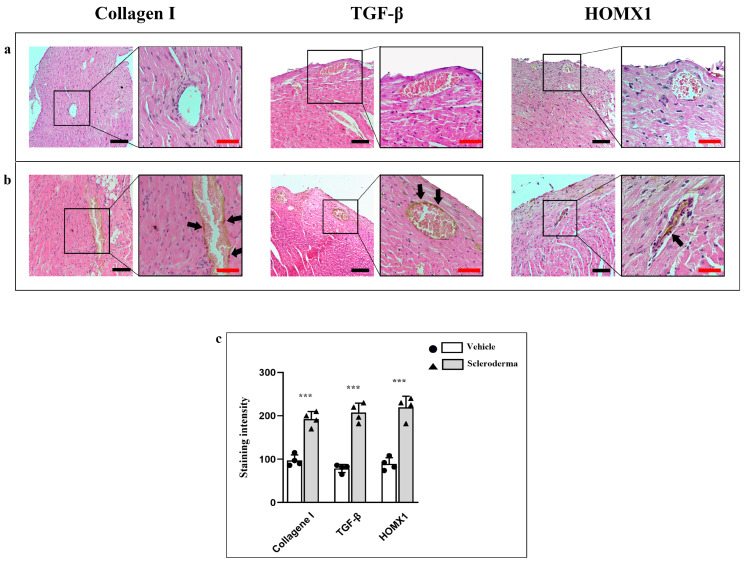
Compound panels of immunoenzymatic images for the evaluation of Collagen I, TGF-β, and HOMX1 in heart sections obtained from vehicle (panel (**a**)) and HOCl mice (panel (**b**)). The black arrows indicate the markers expression around the vessels. The graphic shows the staining intensity and the statistical difference between groups (*** *p* < 0.001) (panel (**c**)). Magnification 20×, black scale bar 50 µm; magnification 40×, red scale bar 25 µm.

α-SMA and vimentin expression were markedly increased in the perivascular region of SSc mice compared to vehicle siblings (Figure 5a,b). Consistent with an increase in fibrotic markers, CD31 expression was reduced in the vessels of HOCl hearts, thus indicating endothelial dysfunction (Figure 5b). Additionally, NRF2 levels were notably elevated, while NOS expression was significantly reduced in HOCl mice, indicating impaired nitric oxide production and further confirming endothelial dysfunction. (Figure 5c,d). The staining intensity for all markers is shown in the graphic (Figure 5e). Moreover, immunoenzymatic results for TGF-β, Collagen I, and HOMX1 have shown a higher expression pattern in HOCL hearts if compared to the vehicle; the staining pattern for all markers has been found to be mainly localized at the peri-vascular level (Figure 6a,b). The staining intensity for these markers is shown in the graphic (Figure 6c). Collectively, these alterations underscore early vascular changes and oxidative stress activation in the sclerodermic myocardium.

## 4. Discussion

In this study, we thoroughly investigated early myocardial involvement in the HOCl-induced murine model of SSc, with a particular focus on the role of oxidative stress, inflammation, endothelial dysfunction, and vascular remodeling. Our data explore the mechanisms that precede the development of overt cardiac fibrosis, highlighting a crucial role for oxidative stress-mediated activation of the NLRP3 inflammasome and its downstream signaling cascade, particularly the induction of endothelial-to-mesenchymal transition (EndMT), as a key driver of early tissue remodeling in the SSc heart. Systemic sclerosis is a multifactorial autoimmune disease sustained by a complex and dynamic interaction among immune cells, endothelial cells, and fibroblasts [22]. To the aim of elucidating the pipeline of early events leading to cardiac fibrosis in SS progression, we evaluated inflammatory and endothelial hallmarks in the HOCl-induced mouse model of SSc at 6 weeks after HOCl injection, when skin and lungs already display an acclaimed fibrotic and inflammatory pattern, a stage comparable to the onset of acclaimed symptoms in SSc patients [23,24,25]. In this model, we observed a distinctive oxidative stress involvement, notably marked by the increased expression of NRF2—a master transcriptional regulator of the antioxidant response pathway—and a concomitant reduction in nitric oxide synthase (NOS) levels, indicative of impaired endothelial homeostasis and dysfunction, at least during the early phases of myocardial involvement.

Oxidative stress is well recognized for its role in activating the NLRP3 inflammasome, a cytoplasmic multiprotein complex that functions as a pivotal sensor of cellular stress and danger-associated molecular patterns [26,27]. Upon activation, this complex facilitates the cleavage and maturation of pro-inflammatory cytokines, including IL-1β, thus amplifying local inflammation and contributing to immune dysregulation and tissue injury [28,29]. In our experimental model, we detected a robust upregulation of NLRP3, as well as its effector molecules, caspase-1 and IL-1β, in myocardial tissue, which strongly supports the activation of the inflammasome-driven inflammatory pathway. This pro-inflammatory activation was paralleled by the increased expression of macrophage markers, including CD68, F4/80, EP29, and EPR1, along with elevated levels of matrix metalloproteinases MMP-3 and MMP-9, indicative of active inflammatory and extracellular matrix remodeling processes [30,31,32,33]. These findings are particularly relevant, as they provide heretofore undocumented evidence that cardiac fibrosis is primed by a pro-fibrotic environment that develops before overt collagen deposition. A central downstream consequence of NLRP3 activation is the induction of EndMT, a phenotypic transition process characterized by the progressive loss of endothelial identity—reflected by the downregulation of CD31—and by the simultaneous acquisition of mesenchymal and fibroblast-like features. This shift was evidenced in our model by a significant upregulation of mesenchymal markers like α-SMA and vimentin [34,35] and by the cytoskeletal remodeling leading to massive production and deposition of extracellular matrix components. Notably, though NLRP3 induces the process of EndMT in SSc extra-cardiac tissues such as the lung and kidney [14,36], we have shown that a similar pathogenic mechanism is active at the cardiac level, thus suggesting that NLRP3-mediated EndMT represents an early and potentially initiating event in myocardial fibrotic transformation.

Consistent with this hypothesis, we identified early structural modifications within cardiomyocytes characterized by the reduced expression of several key structural and adhesion proteins, including dystrophin, dystroglycans, sarcoglycans, talin, and vinculin. These proteins constitute essential components of the dystrophin–glycoprotein complex, which plays a crucial role in maintaining cardiomyocyte mechanical stability and transmitting mechanical and chemical signals across the membrane [7,37,38,39]. The downregulation of these molecules likely compromises membrane integrity, enhances cellular susceptibility to mechanical stress, and promotes inflammatory responses, thereby facilitating the transition to fibrotic remodeling through both direct and indirect pathways.

A central observation in our study is the upregulation of NRF2 (nuclear factor erythroid 2-related factor 2) in myocardial tissue. NRF2 is a master regulator of antioxidant and cytoprotective responses, whose activation promotes the transcription of a wide array of genes involved in redox homeostasis, anti-inflammatory signaling, and cellular defense [40,41,42]. Among its downstream targets, we found a marked increase in HMOX1 (heme oxygenase-1), an enzyme that confers protection against oxidative and inflammatory insults through the degradation of heme and the generation of bioactive metabolites with antioxidant and anti-fibrotic properties [43,44].

The concomitant upregulation of NRF2 and HMOX1 likely reflects an intrinsic protective mechanism aimed at counterbalancing increased oxidative stress and limiting the activation of pro-fibrotic pathways. This interpretation is further supported by the observed elevation of TGF-β, a key cytokine involved in fibroblast activation, extracellular matrix deposition, and the induction of EndMT [45,46]. While TGF-β is a major driver of fibrosis, NRF2 and HMOX1 have been reported to mitigate its downstream effects, suggesting a dynamic interplay between opposing molecular forces during the early stages of myocardial involvement. In this context, the activation of the NRF2/HMOX1 axis may serve to delay or attenuate the fibrotic impact of sustained TGF-β signaling, preserving myocardial architecture in the subclinical phase of the disease. The balance between these protective and pathological pathways likely determines the trajectory of tissue remodeling and the eventual onset of clinically detectable cardiac fibrosis.

As discussed, these mechanisms converge with inflammatory signaling and EndMT, which emerge as critical contributors to the early vascular alterations observed in the myocardium. Considering our findings, the interplay between oxidative stress, inflammation, and endothelial plasticity appears central to the initial phase of cardiac involvement in SSc, providing a mechanistic framework that aligns with the conceptual focus of this study.

Nevertheless, some limitations should be acknowledged. The number of independent biological replicates was limited by ethical and experimental constraints inherent to the use of animal models. Although this may affect the generalizability of the results, variability was minimized by using rigorous technical replicates, standardized procedures, and blinded analyses. Moreover, the HOCl-induced murine model, while well established, does not entirely reproduce the complexity and heterogeneity of human systemic sclerosis. Finally, the analysis was limited to a single experimental time point, which precludes the evaluation of disease progression and late-stage molecular changes. Future studies including larger cohorts, multiple time points, and functional inhibition or genetic silencing of key molecular mediators will be crucial to validate and expand these findings. Despite these limitations, this study provides novel and mechanistically relevant insights into early cardiac involvement in systemic sclerosis and lays the groundwork for future in vivo and translational investigations.

## 5. Conclusions

This study delineates a new and intricate landscape of early myocardial affection in SSc, demonstrating that oxidative stress, NLRP3 inflammasome activation, and EndMT are crucial initiating events in the fibrotic process. These data suggest that the heart, which is classically regarded as a late target in SSc, is already involved very early during SSc through subclinical and dynamic effects. Understanding these mechanisms may have significant implications for identifying early biomarkers and developing targeted therapeutic approaches that modulate inflammation, endothelial plasticity, and oxidative stress, ultimately aiming to prevent irreversible fibrosis. Additional studies will be needed to confirm these results in clinical cases, as well as to further define the therapeutic utility of selective NLRP3 pathway inhibitors or antioxidants in the treatment of SSc-related cardiac fibrosis.

## Figures and Tables

**Table 1:** List of primary antibodies.

Antibody	Species	Dilution	Catalog#/Company
α-sarcoglycan	mouse	1:150	SC-390647 (SantaCruzBiotecnology, Dallas, TX, USA)
γ-sarcoglycan	mouse	1:150	SC-515628 (SantaCruzBiotecnology)
Dystrophin	mouse	1:50	NCLDYS2 (Novocatsra, Wetzlar, Germany)
β-Dystroglycan	mouse	1:250	SC-33702 (SantaCruzBiotecnology)
Talin	mouse	1:500	T3287 (Sigma-Aldrich, St. Louis, MO, USA)
Vinculin	mouse	1:500	V9131 (Sigma-Aldrich)
CD11B	rat	1:250	AB8878 (Abcam, Cambridge, UK)
EP29	rabbit	1:250	AB75853 (Abcam)
EPR	rabbit	1:250	AB208670 (Abcam)
F4/80	rat	1:250	AB6640 (Abcam)
LY6G	rat	1:250	AB210204 (Abcam)
MMP-3	rabbit	1:250	AB52915 (Abcam)
MMP-9	rabbit	1:250	AB283575 (Abcam)
Caspase-1	rabbit	1:100	STJ92017 (St John’s, Anaheim, CA, USA)
IL-1β	mouse	1:250	SC-52012 (SantaCruzBiotecnology)
NLRP-3	rabbit	1:250	AB263899 (Abcam)
α-SMA	mouse	1:500	SAB5700835 (Sigma-Aldrich)
CD31	rabbit	1:100	AB222783 (Abcam)
Vimentin	rabbit	1:1500	100619 (GeneTex, Irvine, CA, USA)
NOS	mouse	1:200	SC-7271 (SantaCruzBiotecnology)
NRF-2	Rabbit	1:100	STJ97502 (St John’s)
HOMX1	Rabbit	1:150	SC-390991 (SantaCruzBiotecnology)
TGF-β	mouse	1:250	SC-130348 (SantaCruzBiotecnology)
Collagen I	rabbit	1:100	PA1-26204 TermoFisher Scientific, Waltham, MA, USA

**Table 2:** List of secondary antibodies and fluorochromes.

Antibody	Species	Dilution	Catalog#/Company
Fluorescein anti-rat	goat	1:100	AP136F (Chemicon, Bangalore, India)
FITC anti-rabbit	goat	1:100	111-096-045 (Jackson ImmunoResearch, West Grove, PA, USA)
Alexa Fluor^TM^	goat	1:800	A-11001 (Invitrogen, Waltham, MA, USA)

## Data Availability

All data were included in the article.
